# Age-related change in sit-to-stand power in Japanese women aged 50 years or older

**DOI:** 10.1186/1880-6805-33-26

**Published:** 2014-08-16

**Authors:** Hiroaki Kanehisa, Tetsuo Fukunaga

**Affiliations:** 1 National Institute of Fitness and Sports in Kanoya, 1 Shiromizu, Kanoya, Kagoshima 891-2393, Japan

**Keywords:** Field test, Motor coordination, Ultrasonography

## Abstract

**Background:**

This study examined whether the age-related change in power, calculated from the score of a sit-to-stand (STS) test, corresponds to those in knee extension torque and leg lean tissue mass in Japanese women aged 50 years or older.

**Findings:**

Time for a 10-times-repeated STS test and knee extension torque were determined in 556 Japanese women aged 50 to 94 years. STS power was calculated using an equation reported previously. In addition, leg lean tissue mass was estimated using muscle thicknesses determined at thigh and lower leg. STS power, knee extension torque, and lean tissue mass were negatively correlated to age. STS power and knee extension torque, expressed as the percentages of the mean value of the corresponding variable for the subjects aged 50 to 54 years were lower than that of lean tissue mass in the subjects aged 60 years or over, and were similar in those aged under 75 years. However, the relative value of STS power was lower than that of knee extension torque in the subjects aged over 75 years.

**Conclusions:**

In Japanese women aged 50 to 74 years, STS power can be a convenient measure for assessing the age-related decline in knee extension torque, but not for leg lean tissue mass. At over 75 years old, the magnitude of the age-related decline in STS power does not parallel to that in the force generation capability of knee extensor muscles.

## Findings

### Background

The force generation capacity of skeletal muscles decreases with aging [1,2]. Among muscles in the limbs, the knee extensor muscles show large age-related reduction [2,3]. This directly increases the difficulty of performing activities of daily living (ADLs), such as walking, standing up, and climbing steps [4,5]. For the elderly, therefore, it is essential to establish a method conveniently assessing the magnitude of functional disability for designing exercise or rehabilitation programs. As an approach to this, some researchers have attempted to examine the applicability of a sit-to-stand (STS) test for assessing the force and/or power generation capability of knee extensor muscles in an elderly population [6,7]. Takai *et al.*[6] found that power, calculated from the performance of the STS test, can be a convenient measure to assess the cross-sectional area and strength of the knee extensors in the elderly. However, no studies have examined age-related change in STS power. Furthermore, it is unknown whether the age-related change in STS power corresponds to those in knee extensor strength and muscle mass of the leg. The present study aimed to examine these issues.

### Methods

#### ***Subjects***

Five hundred and fifty-six Japanese women aged 50 to 94 years voluntarily participated in this study. None of the subjects was or had been an athlete. The subjects were classified into seven age groups with intervals of 5 years from the age of 50 years: subjects aged 50 to 54 years (G_50-54_), 55 to 59 years (G_55-59_), 60 to 64 years (G_60-64_), 65 to 69 years (G_65-69_), 70 to 74 years (G_70-74_), 75 to 79 years (G_75-79_), and 80 years and over (G_80+_). The physical characteristics of each subject group are summarized in Table 1. Fifteen of the subjects in G_80+_ had received care under an insurance system. None had a history of diseases such as CNS disorder or were unable to go out unsupported and refrained from going out alone because of decreased physical capacity. All subjects could successfully complete the prescribed procedures for the STS test and knee extension torque measurements. This study was approved by the Ethics Committee of the Graduate School of Arts and Sciences, University of Tokyo, and was consistent with the institutional ethical requirements for human experimentation in accordance with the Declaration of Helsinki. The subjects were fully informed of the purpose and risks of the experiment and gave their written informed consent.

**Table 1:** Physical characteristics of the subjects

	**Age group**						
**Variables**	**G**_**50-54**_** n = 32**	**G**_**55-59 **_**n = 56**	**G**_**60-64 **_**n = 112**	**G**_**65-69 **_**n = 151**	**G**_**70-74 **_**n = 126**	**G**_**75-79 **_**n = 57**	**G**_**80+ **_**n = 22**
Age, years	52.8 (1.3)	57.6 (1.6)	62.8 (1.5)	67.4 (1.4)	72.4 (1.4)	77.1 (1.4)	86.0 (4.3)
Height, m	1.537 (0.048)	1.542 (0.048)	1.515 (0.049)	1.502 (0.050)	1.495 (0.055)	1.471 (0.059)	1.429 (0.062)
Thigh length, m	0.350 (0.015)	0.351 (0.017)	0.343 (0.017)	0.341 (0.018)	0.342 (0.020)	0.336 (0.017)	0.332 (0.017)
Lower leg length, m	0.339 (0.018)	0.341 (0.017)	0.335 (0.017)	0.333 (0.017)	0.334 (0.018)	0.328 (0.016)	0.328 (0.015)
Body mass, kg	53.8 (6.1)	55.7 (7.2)	54.2 (6.5)	53.4 (6.9)	52.3 (6.9)	50.5 (6.7)	48.1 (6.6)
BMI, kg/m^2^	22.8 (2.3)	23.5 (3.2)	23.7 (3.0)	23.7 (3.0)	23.4 (2.7)	23.3 (2.7)	23.6 (3.0)

Values are means (SDs).

BMI, body mass index.

G_50-54_, the subjects aged 50 to 54 years; G_55-59_, the subjects aged 55 to 59 years; G_60-64_, the subjects aged 60 to 64 years; G_65-69_, the subjects aged 65 to 69 years; G_70-74_, the subjects aged 70 to 74 years; G_75-79_, the subjects aged 75 to 79 years; G_80+_, the subjects aged 80 years and over.

#### ***Measurements of muscle thickness (MT)***

MTs at thigh anterior and posterior and lower leg anterior and posterior on the right side of the body were determined using a real time B-mode ultrasound apparatus (SSD-500, Aloka Co., Tokyo, Japan) in accordance with the procedure used in a prior study [8]. The MTs at the four sites were used to estimate the lean tissue mass (LTM) of the leg, being calculated using the prediction equation developed by Takai *et al.*[8]: LTM (kg) = 0.01464 × {thigh MT (anterior + posterior) × thigh length (cm^2^) + lower leg MT (anterior + posterior) × lower leg length (cm^2^)} ‒ 2.767 (R^2^ = 0.96, SEE = 0.3 kg), where the thigh length and lower leg length represent the distance from the greater trochanter to the popliteal crease and the distance from the popliteal crease to the lateral malleolus, respectively. The repeatability of the MT measurements was tested by determining 10 young adults twice on separate days. There were no significant inter-day differences in limb length and MT measurements, with intraclass correlation coefficients (ICCs) of 0.997 for the thigh length and lower leg length, and of 0.860 to 0.970 for the MTs at the four sites. The mean value of coefficient of variation (CV) was 0.2% for the thigh length and lower leg length, and 1.1 to 2.7% for the MTs.

#### ***Measurements of knee extension torque (KETQ)***

The maximal voluntary isometric KETQ of the right leg was determined using a specially designed myometer (VTK-002R/L, Vine, Japan) in accordance with the procedure described in a prior study [6]. Prior to the test, the subjects were asked to perform sub-maximal contractions two to three times to familiarize themselves with the test procedure. In the maximal test, the subjects executed a 2- to 3-s maximal voluntary contraction two times, with at least 1 min of rest between the trials. If the difference between the two values was more than 10%, the torque was measured once more. The highest torque value of the two or three measurements was adopted.

#### ***Sit-to-stand (STS) test***

In accordance with the procedure described in a prior study [6], the time required to perform a 10-times-repeated STS test on a steel molded chair (0.40 m height and 0.36 m depth) was determined. In the measurements, the subjects were asked to stand up from a sitting position and then to sit down 10 times as fast as possible. The time (STS-T) was recorded using a stopwatch to the nearest 10th of a second. Prior to the test trials, the subjects were asked to perform practice trials with submaximal effort to confirm the position of the chair and to familiarize themselves with the test procedure. After 1 min of rest following completion of the practice session, the subjects performed the STS test two times with an interval of 1 min between the trials. The faster time was adopted for the individual data. STS power (STS-P) was calculated using the following equation [6]: STS-P(W) = {thigh length (m) + lower leg length (m) - 0.4 (m)} × body mass (kg) × g (m/s^2^) × 10/STS-T (s), where the 0.4 (m) and g (m/s^2^) represent the height of the chair and acceleration due to gravity of 9.8 m/s^2^, respectively.

The repeatability of KETQ and STS measurements was tested by examining 17 men and 35 women aged 35 to 76 years twice on separate days. There were no significant differences between the first and second measurements in KETQ (90.5 ± 31.4 Nm *vs.* 89.9 ± 33.6 Nm), STS-T (10.5 ± 2.3 s *vs.* 10.5 ± 2.2 s), and STS-P (162.9 ± 63.2 W *vs.* 161.8 ± 61.2 W). ICC was 0.963 for KETQ, 0.877 for STS-T, and 0.977 for STS-P. The mean value of %CV was 5.9% for KETQ, 5.6% for STS-T, and 5.2% for STS-P. In each of the three variables, the relative difference between the first and second measurements was not significantly associated with age: r = -0.072 (n.s.) for KETQ, r = -0.029 (n.s.) for STS-T, and r = 0.027 (n.s.) for STS-P. On the basis of these results, we considered that the KETQ and STS measurements have high repeatability.

#### ***Statistics***

Descriptive values are presented as means ± SDs. To compare the rates of age-related changes among LTM, KETQ, and STS-P, we calculated the percentages of their values for individual to the mean value of the corresponding variable for G_50-54_. The relative values of LTM, KETQ, and STS-P were referred to as %LTM, %KETQ, and %STS-P, respectively. A one-way analysis of variance (ANOVA) with a Tukey test was used to test the differences among %LTM, %KETQ, and %STS-P within the same age group. A simple linear regression analysis was used to calculate the coefficients of correlations between age and each of %LTM, %KETQ, and %STS-P. The probability level for statistical significance was set at *P* <0.05.

### Results

**Figure 1:**
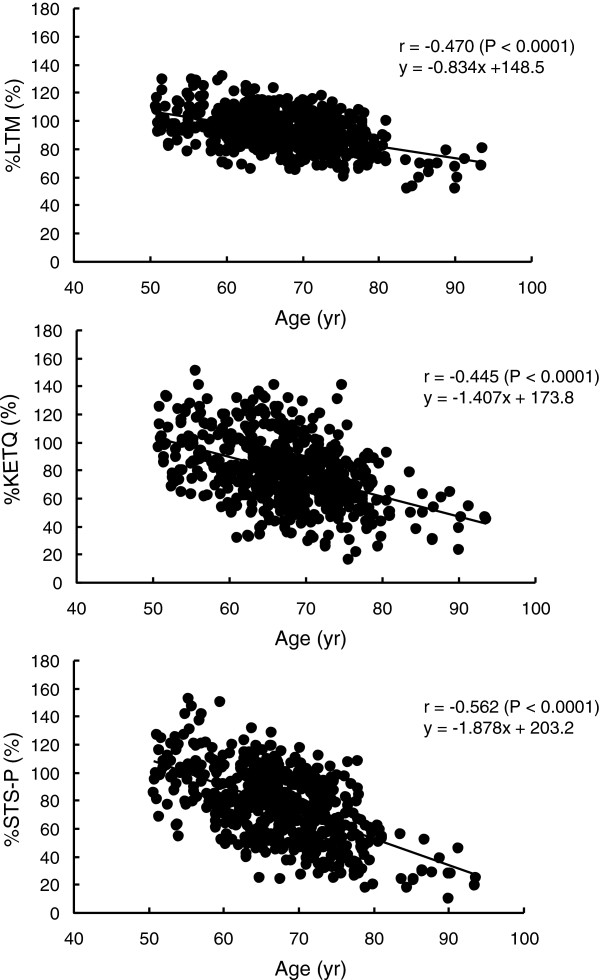
Relationship between age and each of %LTM (upper), %KETQ (middle), and %STS-P (lower).

**Figure 2:**
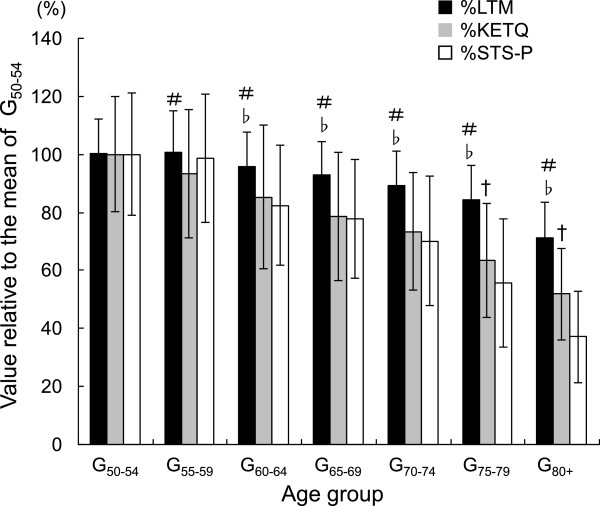
**Age-related changes of the value relative to the mean value of the subjects aged 50 to 54 years in the estimated lean tissue mass (%LTM), knee extension torque (%KETQ), and sit-to-stand power (%STS-P).** The presented values are the mean and SD in each of the age groups, which were classified with intervals of 5 years. # indicates that the mean value of %LTM is significantly (*P* <0.05) higher than that of %KETQ. ♭ indicates that the mean value of %LTM is significantly (*P* <0.05) higher than that of %STS-P. † indicates that the mean value of %KETQ is significantly higher (*P* < 0.05) than that of %STS-P.

Descriptive data on the measured variables for each age group are summarized in Table 2. Each of %LTM, %KETQ, and %STS-P was negatively correlated to age (Figure 1). The rate of decline in the regression line (that is, regression coefficient) for the relationship was high in the order of %STS-P, %KETQ, and %LTM. Figure 2 shows a comparison among %LTM, %KETQ, and %STS-P within the same age group. In the G_55-59_ group, %LTM was similar to %STS-P, but was significantly higher than %KETQ. In the other age groups, %LTM showed significantly higher values than %KETQ and %STS-P. There were no significant differences between %KETQ and %STS-P in the age groups in which the ages of the subjects were under 75 years. In G_75-80_ and G_80+_, however, %STS-P was significantly lower than %KETQ.

**Table 2:** Descriptive data on muscle thickness (MT), knee extension torque (KETQ), estimated lean tissue mass (LTM), time for sit-to-stand test (STS-T), and sit-to-stand power (STS-P)

	**Age group**						
**Variables**	**G50-54 n = 32**	**G55-59 n = 56**	**G60-64 n = 112**	**G65-69 n = 151**	**G70-74 n = 126**	**G75-79 n = 57**	**G80+ n = 22**
MT, cm							
Thigh anterior	4.4 (0.6)	4.3 (0.6)	4.1 (0.6)	3.8 (0.5)	3.7 (0.5)	3.4 (0.4)	3.0 (0.5)
Thigh posterior	5.7 (0.5)	5.7 (0.7)	5.8 (0.7)	5.7 (0.7)	5.5 (0.8)	5.5 (0.7)	4.9 (0.8)
Lower leg anterior	2.6 (0.2)	2.6 (0.3)	2.7 (0..2)	2.6 (0.3)	2.5 (0.3)	2.5 (0.2)	2.3 (0.3)
Lower leg posterior	6.0 (0.4)	6.0 (0.5)	6.0 (0.5)	6.0 (0.5)	5.9 (0.4)	5.9 (0.5)	5.4 (0.5)
LTM, kg	6.7 (0.8)	6.7 (1.0)	6.4 (0.8)	6.2 (0.8)	5.9 (0.8)	5.6 (0.8)	4.8 (0.8)
KETQ, Nm	90.5 (17.9)	84.4 (20.0)	77.1 (22.5)	70.9 (20.0)	66.5 (18.3)	57.4 (17.7)	47.0 (14.2)
STS-T, s	9.8 (2.0)	10.4 (2.0)	11.8 (3.2)	12.2 (3.6)	13.9 (5.1)	16.6 (6.1)	24.0 (9.4)
STS-P, W	159.8 (33.8)	157.3 (35.3)	131.7 (33.1)	124.4 (32.8)	111.9 (35.6)	89.0 (35.1)	59.2 (25.1)

Values are means (SDs).

### Discussion

The observed differences among %STS-P, %KETQ, and %LTM indicate that, in Japanese women aged 50 years or over, the age-related declines in the isometric and dynamic performances of the lower body are greater than that in the lean tissue mass. At the same time, the current result implies that, when STS power is used as an alternative index of leg LTM, it overestimates the age-related change in leg LTM.

%KETQ and %STS-P were similar in the age groups in which the ages of the subjects were under 75 years. This indicates that STS power can be a convenient measure representing the age-related change in KETQ until the age of 75 years. In G_75-80_ and G_80+_, however, %STS-P was lower than %KETQ. Skelton *et al.*[9] reported that the age-related decline of explosive power was more rapid than that of knee extensor strength in men, but not significantly so in women aged 65 to 89 years. In addition, Yamauchi *et al.*[10] reported that age-related decline in the lower maximum power output of multi-joint movement in elderly women did not depend on that in the intrinsic shortening velocity of muscle action, but largely on the reduction in force generating capacity. Considering these findings, it seems that the age-related declines in %KETQ and %STS-P would be similar regardless of age. However, the observed difference between %STS-P and %KETQ in G_75-79_ and G_80+_ refutes this and implies that the age-related decline in STS power after the age of 75 years may involve factors other than that in force generation capability.

Sit-to-stand task requires that the individual exerts forces with appropriate magnitude and timing [11]. Aging affects motor coordination strategies during sit-to-stand [12]. Linderman *et al.*[11], who examined the coordination of strength exertion during the chair rise movement in very old people, reported that that the strongest predictor of total time for the standing up task was the time elapsed in the stabilization phase after the rising phase. In addition, Gross *et al.*[13] indicated that knee extensor strength was less important than hip strength to chair-rise biomechanics in elderly women. Taking these aspects into account, it seems that the observed differences between %STS-P and %KETQ in the subjects aged 75 years or older would be attributed to the possible age-related change in motor coordination strategies during the STS task.

### Conclusion

In Japanese women aged 50 to 74 years, power, estimated from the performance of STS test, can be a convenient measure for assessing the age-related decline in knee extension torque, but it declines more rapidly than leg lean tissue mass. After the age of 75 years, the magnitude of the age-related decline in STS power does not parallel to that in the force generation capability of knee extensor muscles. The current results were obtained from the Japanese women aged 50 years or older, who could successfully complete the prescribed procedure for the STS test. Therefore, the present study cannot provide information concerning the age-related profiles of knee extension torque and/or leg lean tissue mass in individuals with impaired function in chair rise. In addition, there is a possibility that, if frail elderly persons with a greater difficulty for performing standing task are involved, the age-related changes in STS power, knee extension torque, and leg lean tissue mass might be different from those observed here. Further investigation is needed to clarify these issues.

## Abbreviations

G_50-54_: Subjects aged 50 to 54 years; G_55-59_: Subjects aged 55 to 59 years; G_60-64_: Subjects aged 60 to 64 years; G_65-69_: Subjects aged 65 to 69 years; G_70-74_: Subjects aged 70 to 74 years; G_75-79_: Subjects aged 75 to 79 years; G_80+_: Subjects aged 80 years and over; KETQ: Knee extension torque; LTM: Lean tissue mass; MT: Muscle thickness; STS test: Sit-to-stand test; STS-P: Power calculated from the score of a 10-times- repeated STS test; STS-T: Time for a 10-times-repeated STS test; %STS-P: STS power value relative to the mean value of the subjects aged 50 to 54 years; %LTM: LTM value relative to the mean value of the subjects aged 50 to 54 years; %KETQ: KETQ value relative to the mean value of the subjects aged 50 to 54 years.

## Competing interests

The authors declare that they have no competing interests.

## Authors’ contributions

HK participated in study design, helped acquire funding from the Ministry of Education, Culture, Sports, Science and Technology, helped coordinate research activities, performed statistical analysis, and drafted the manuscript. TF participated in study design and coordination, and drafted the manuscript. Both authors read and approved the final manuscript.
